# Can Patient Education Lead the Way? Head‐To‐Head Comparison of EXACT and CERT for Early Recognition of Acute COPD Exacerbations

**DOI:** 10.1002/resp.70170

**Published:** 2025-11-30

**Authors:** Rainer Gloeckl, Paul W. Jones, Daniela Kroll, Inga Jarosch, Tessa Schneeberger, Jing Claussen, Paul Schmidt, Claus F. Vogelmeier, Klaus Kenn, Andreas Rembert Koczulla

**Affiliations:** ^1^ Institute for Pulmonary Rehabilitation Research Schön Klinik Berchtesgadener Land Schoenau am Koenigssee Germany; ^2^ Philipps‐University Marburg German Center for Lung Research (DZL) Universities of Giessen and Marburg Lung Center (UGMLC) Marburg Germany; ^3^ St. George's University of London London UK; ^4^ Research and Development GlaxoSmithKline Uxbridge UK; ^5^ Medical Affairs GlaxoSmithKline Munich Germany; ^6^ Statistical Consulting for Science and Research Berlin Germany; ^7^ Paracelsus Medical University Salzburg Austria

**Keywords:** AECOPD, chronic obstructive pulmonary disease, COPD exacerbation recognition tool, diary, exacerbation of chronic pulmonary disease tool, self‐monitoring

## Abstract

**Background and Objective:**

Acute exacerbations of COPD (AECOPD) worsen outcomes, yet they are often underreported. The 14‐item EXACT diary has been validated for trial use—the five‐item CERT checklist is patient‐centered. We compared the two tools' accuracy and early‐warning performance for AECOPD.

**Methods:**

This prospective study examined 63 COPD patients who completed EXACT and CERT daily. Two metrics were used for CERT: total score and binary (positive if ≥ 2 items were rated as moderate/severe). AECOPD patients were bootstrap matched to a non‐exacerbator. Linear mixed‐effects models assessed score differences on the day of AECOPD diagnosis and the previous days.

**Results:**

Twelve patients developed clinically confirmed AECOPD. On the day of AECOPD diagnosis, all tools discriminated AECOPD (AUC: EXACT 0.90, CERT total score 0.88, and CERT binary 0.87; *p* < 0.01). At optimal thresholds, sensitivities ranged from 81% to 87%, and specificities ranged from 93% to 95%. One day before the AECOPD diagnosis, the CERT total score (5.9 vs. 1.4; *p* = 0.006) and the CERT binary outcome (70.4% vs. 9.3%; *p* = 0.009) significantly distinguished impending exacerbations. However, the EXACT score did not (39.6% vs. 34.3%; *p* = 0.186). The CERT total score yielded an AUC of 0.86 (sensitivity 83%, specificity 86%), and the binary rating achieved a specificity of 91% and a sensitivity of 70% one day before diagnosis.

**Conclusion:**

Both EXACT and CERT can accurately detect AECOPD at the time of diagnosis. However, patients were able to recognise worsening symptoms one day before an AECOPD diagnosis when using the CERT.

**Trial Registration:**

clinicaltrials.gov (NCT04140097)

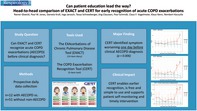

## Graphical abstract

Daily head‐to‐head comparison of EXACT vs. CERT for early detection of acute COPD exacerbations. Both tools accurately identified exacerbations at diagnosis (AUC≈0.9), but CERT detected worsening one day earlier (*p* = 0.006). CERT's simplicity and free access support rapid patient self‐monitoring and may enable timely intervention.

**See related** editorial

## 1 Introduction

Acute exacerbations of chronic obstructive pulmonary disease (AECOPD) are major drivers of disease progression, increased healthcare utilisation, and mortality [1]. Even a single exacerbation is associated with accelerated lung function decline, impaired exercise capacity, and increased risk of cardiovascular events and death [2]. Despite their clinical relevance, diagnosing exacerbations remains challenging due to variability in symptom presentation and the lack of reliable biomarkers [3]. Clinical criteria, such as those defined by Anthonisen et al. [4], emphasise changes in dyspnea, sputum volume, and purulence, but have limitations in sensitivity and applicability to modern COPD care [3]. Furthermore, a critical issue in COPD management is the high rate of AECOPD underreporting by patients. Across multiple studies and countries, between 40% and 90% of exacerbations remain unreported [5, 6, 7]. Patients often ‘wait and see’, self‐medicate, or misattribute symptoms to their baseline disease or common infections. These unreported events have been linked to a longer recovery period and a shorter time to the next exacerbation. Timely reporting and treatment, on the other hand, are significantly associated with faster recovery [8]. A recent international survey also found substantial variability in how COPD patients identify exacerbations, pointing to inconsistent self‐recognition of these events [9]. This heterogeneity underscores the need for structured educational tools to standardise patients' interpretation of early exacerbation signs and empower them to recognise and act on symptom worsening.

The EXAcerbations of Chronic Pulmonary Disease Tool (EXACT) is a 14‐item patient‐reported diary developed to detect and quantify exacerbations [10, 11]. It is validated and has regulatory acceptance for use in drug development studies [3]. However, EXACT is rather complex, and the daily diary format may limit its feasibility for widespread and regular use in clinical practice.

**FIGURE 1:**
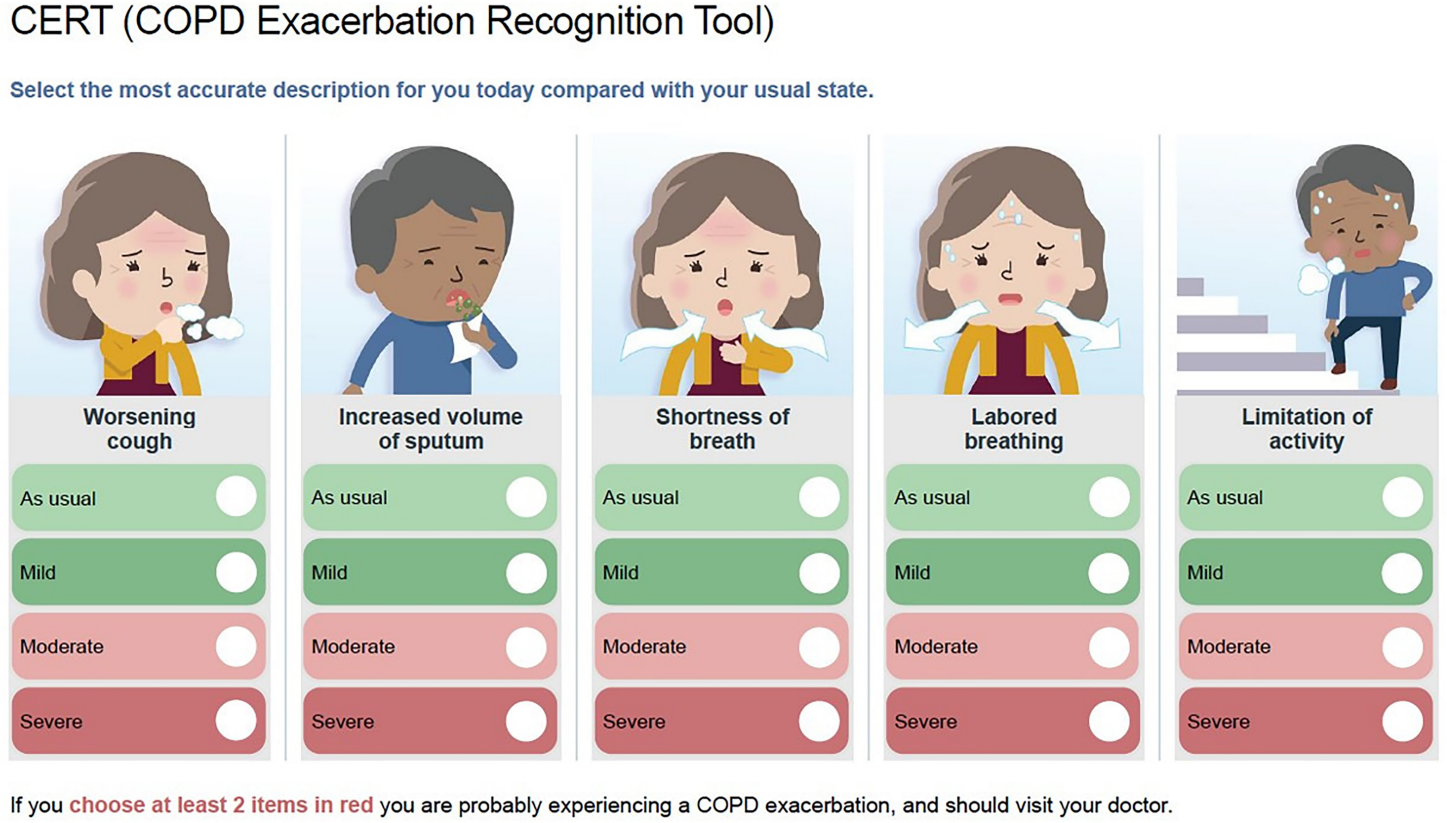
COPD Exacerbation Recognition Tool (CERT), freely available at www.gaapp.org/cert, by GSK.

The COPD Exacerbation Recognition Tool (CERT), a simple, five‐item tool was recently developed to help patients recognise AECOPD and advise them when to seek medical advice [12]. CERT was developed as an educational aid, rather than a daily diary, to improve awareness of and reporting of symptoms. Patients are instructed to refer to it for guidance when they notice a deterioration in their condition. It uses patient‐centred language and pictograms to highlight the key changes most associated with exacerbations: breathlessness, sputum volume and activity limitation (Figure 1). While both CERT and EXACT target the early signs of AECOPD, EXACT is a validated daily diary used in clinical trials, whereas CERT is an episodic educational tool that helps patients quantify symptom deterioration. To date, no study has directly compared EXACT and CERT in the same population. Therefore, we aimed to compare the two tools' accuracy and potential as early predictors of an AECOPD.

## 2 Methods

### 2.1 Study Design and Setting

This study was part of the *Predictors of Acute COPD Exacerbation* (PACE) study, a prospective, monocentric observational study aimed at identifying predictors of acute exacerbations in COPD patients (e.g., biomarkers, physiological measures, and patient‐reported symptoms). The PACE study enrolled COPD patients between February 2020 and April 2024 during a 3‐week inpatient pulmonary rehabilitation program at the Schoen Klinik Berchtesgadener Land (Schoenau am Koenigssee, Germany). From February 2020 through June 2023, patients completed the EXACT daily [13]. Beginning in July 2023, the newly developed CERT was added daily alongside the EXACT until April 2024. This study amendment aimed to evaluate the ability of each tool to detect clinician‐confirmed AECOPD on the day of diagnosis and their potentials to predict an impending exacerbation even before AECOPD diagnosis.

The PACE study, including this amendment, was approved by the Ethical Committee of the Philipps University Marburg, Germany (approval No. 61/19) and registered at clinicaltrials.gov (NCT04140097) [13].

### 2.2 Participants and Eligibility Criteria

COPD patients were recruited consecutively upon admission to the inpatient pulmonary rehabilitation unit. Eligible participants were aged ≥ 40 years with a confirmed diagnosis of COPD (GOLD stage II to IV; post‐bronchodilator FEV_1_/FVC < 0.7 and FEV_1_ ≤ 80% predicted), able to participate in rehabilitation and complete daily symptom diaries, and had provided written informed consent. Patients were excluded if they were experiencing an AECOPD at admission, had a primary diagnosis of asthma or another significant non‐COPD respiratory condition, or were unable to complete study procedures (e.g., due to cognitive impairment or language barrier).

### 2.3 EXACT and CERT Administration

During the PACE study amendment period, all newly enrolled and continuing participants were instructed in the administration of both EXACT and CERT, regardless of exacerbation status, to ensure uniform data collection. Table 1 provides an overview of the key principles of these two tools. Participants used pen‐and‐paper diaries to complete both tools daily in the evening. This study used a German‐language CERT version with a slightly modified instruction set. Instead of asking patients to compare their symptoms to their ‘usual state’ (as in the original validation study), patients rated symptom deterioration over ‘the last two days’. This change was introduced to harmonise reporting across time points and reduce recall bias during daily use.

**TABLE 1:** Key features of the Exacerbations of Chronic Obstructive Pulmonary Disease tool (EXACT) and the COPD Exacerbation Recognition Tool (CERT).

Tool	Items	Intended use	Frequency	Scoring	Licensing
EXACT	14	Research diary	Daily (mandatory)	0–100 points total score	Proprietary
CERT	5	Educational checklist	As‐needed (educational)	Rating ≥ 2 items as moderate/severe or 0–15 points total score	Free, open access

### 2.4 Exacerbations of Chronic Obstructive Pulmonary Disease Tool (EXACT)

The EXACT is a 14‐item patient‐reported outcome measure designed to quantify and track COPD symptom severity and exacerbation status [14]. Each item is scored on a 5‐ or 6‐point ordinal scale and converted to a 0–100 total score (higher = more severe symptoms). EXACT was developed following Food and Drug Administration (FDA) guidance for patient‐reported outcome instruments [10]. Its validity for both exacerbation detection and severity grading has been demonstrated in real‐world cohorts [15]. A ≥ 12‐point increase above baseline for two consecutive days or a ≥ 9‐point increase for 3 days defines an EXACT‐based exacerbation event. The EXACT is a copyright‐protected, instrument developed by the COPD Foundation and distributed by Evidera Inc. We obtained a license from Evidera Inc. to ensure compliant administration and scoring of the EXACT for this study.

### 2.5 COPD Exacerbation Recognition Tool (CERT)

The CERT is a five‐item symptom checklist that guides patients on when to seek medical advice during symptom worsening (Figure 1). CERT items include worsening cough, increased sputum volume, shortness of breath, laborious breathing, and limited activities and can be rated as usual, mild, moderate, or severe compared to the usual state. A positive score is indicated if two or more items are rated as moderate or severe [12]. This suggests that patients are probably experiencing an AECOPD and should visit a doctor. Recently, a second approach to interpreting CERT was introduced and adopted in our analyses. This approach used a total score ranging from 0 (as usual) to 3 (severe) per item, resulting in a total score ranging from 0 to 15 [16]. The CERT is free for clinicians and patients to use and can be downloaded in various languages at: www.gaapp.org/cert.

Although the CERT was originally developed as an episodic checklist to be used only when symptoms worsen, we applied it to daily use in this study to enable a direct comparison with the EXACT. This approach ensured methodological consistency and allowed for an accurate comparison of the two tools' early‐warning potential.

### 2.6 Clinical Definition and Assessment of AECOPD

AECOPD events were defined per the original PACE protocol and Anthonisen criteria requiring the presence of at least two of three cardinal symptoms, including an increase or new onset of dyspnea, sputum production, and sputum purulence [4]. During inpatient rehabilitation, an experienced pulmonologist conducted daily clinical evaluations including physical examination with auscultation, and when indicated spirometry, blood gas analyses, laboratory tests (e.g., CRP), or chest imaging. AECOPD onset date was the first day the pulmonologist confirmed symptom worsening meeting these criteria. The day of clinical diagnosis was recorded as the reference for cross‐sectional detection analyses. Pulmonologists were blinded to EXACT and CERT ratings to avoid incorporation bias.

## 3 Statistical Analysis

Analyses focused on two primary objectives: (1) cross‐sectional detection of AECOPD on the day of clinical AECOPD diagnosis using EXACT and CERT; (2) prediction of AECOPD before clinical diagnosis. For both objectives, patients who developed an AECOPD during inpatient stay were compared with patients who did not develop an AECOPD.

### 3.1 Random Matching With Bootstrapping

To compare patients with and without AECOPD at the time of exacerbation, we applied a bootstrap procedure. In each of 1000 iterations, we drew, with replacement, 12 individuals from the AECOPD cohort (*n* = 12) and 12 from the larger non‐AECOPD pool (*n* = 51). Each selected control was then paired with an AECOPD patient, and an artificial AECOPD date was generated for the control by mirroring its partner's trajectory. Repeating this resampling ensured that every control could appear multiple times, allowing the entire control population to contribute to the final estimates while maintaining balanced sample sizes across iterations.

### 3.2 Modelling EXACT and CERT Scores

Between‐group differences in EXACT and CERT total scores at each time point around AECOPD diagnosis were evaluated with linear mixed‐effects models. Time and group were specified as categorical fixed effects, the baseline score was entered as a continuous covariate, and patient ID was included as a random effect. For the binary CERT ‘≥ 2 items’ criterion, a generalised linear mixed‐effects model with a logit link was used. Results were reported as estimated marginal means (EMMs) with standard errors and 95% confidence intervals, or as odds ratios with 95% confidence intervals, and statistical significance was defined as *α* = 0.05. Receiver operating characteristic (ROC) curves were generated separately for the EXACT total score and the CERT total score. Areas under the curve (AUC) were calculated, with 95% confidence intervals estimated via nonparametric methods. Sensitivity, specificity, positive predictive value (PPV), and negative predictive value (NPV) were calculated based on the optimal threshold obtained from the ROC analysis (Youden‐Index). Only patients with valid baseline values were considered. Missing values at other time points were treated as missing.

## 4 Results

A total of 85 COPD patients were recruited in the PACE study between July 2023 and April 2024. We excluded 14 patients due to missing EXACT or CERT data. Furthermore, we excluded eight patients (two AECOPD and six non‐AECOPD patients) because they provided consistently high scores instead of rating symptom deterioration over the last two days on the CERT, which suggests a potential misunderstanding of the tool. Two authors (RG and PS) independently evaluated these patient data and reached a consensus. In the end, our analyses included 63 COPD patients. During the three‐week observational phase, 12 patients developed an AECOPD, while 51 patients did not (see Table 2 for baseline characteristics). All AECOPD patients were treated with oral corticosteroids for 5 days at a dose of 40 mg/day, and nine of them (75%) also received antibiotic treatment. Since patients were already in an inpatient pulmonary rehabilitation center, none required referral to an acute hospital.

**TABLE 2:** Baseline characteristics.

	No AECOPD	AECOPD
*n*	51	12
Age, years	68 (61, 77)	71 (68, 74)
Female, *n* (%)	20 (39%)	7 (58%)
BMI, kg/m^2^	22.1 (20.1, 26.0)	19.0 (16.7, 25.0)
Smoking status, current/former/never, *n* (%)	5 (10%)/43 (84%)/3 (6%)	3 (25%)/9 (75%)/0 (0%)
Number of all previous AECOPDs	1 (0, 4)	4 (1, 7)
Number of AECOPDs (< 12 months)	1 (0, 1)	1 (0.5, 2)
Number of hospitalizations due to AECOPD (< 12 months)	0 (0, 2)	1.5 (0, 4)
LABA, *n* (%)	48 (94%)	12 (100%)
LAMA, *n* (%)	50 (98%)	12 (100%)
ICS, *n* (%)	29 (57%)	10 (83%)
OCS, *n* (%)	6 (12%)	4 (33%)
FEV_1_, %predicted	37 (29, 50)	36 (28, 44)
FEV_1_/FVC	48.9 (41.9, 58.3)	51.0 (44.2, 54.3)
RV, %predicted	224 (186, 261)	231 (205, 262)
pO_2_, mmHg	64.4 (58.3, 69.1)	56.2 (51.8, 58.2)
6MWD, m	367 (265, 415)	328 (269, 363)

*Note*: Data are presented as median and interquartile range or number and percentage.

Abbreviations: 6MWD—6‐min walk distance; AECOPD—acute exacerbation of chronic; obstructive pulmonary disease; BMI—body mass index; FEV_1_—forced expiratory volume in 1 s; FVC—forced vital capacity; ICS—inhaled corticosteroids; LABA—long‐acting beta‐agonist; LAMA—long‐acting muscarinic antagonist; OCS—oral corticosteroids; RV—residual volume.

### 4.1 Cross‐Sectional Discrimination of AECOPD on the Day of Clinical Diagnosis

**FIGURE 2:**
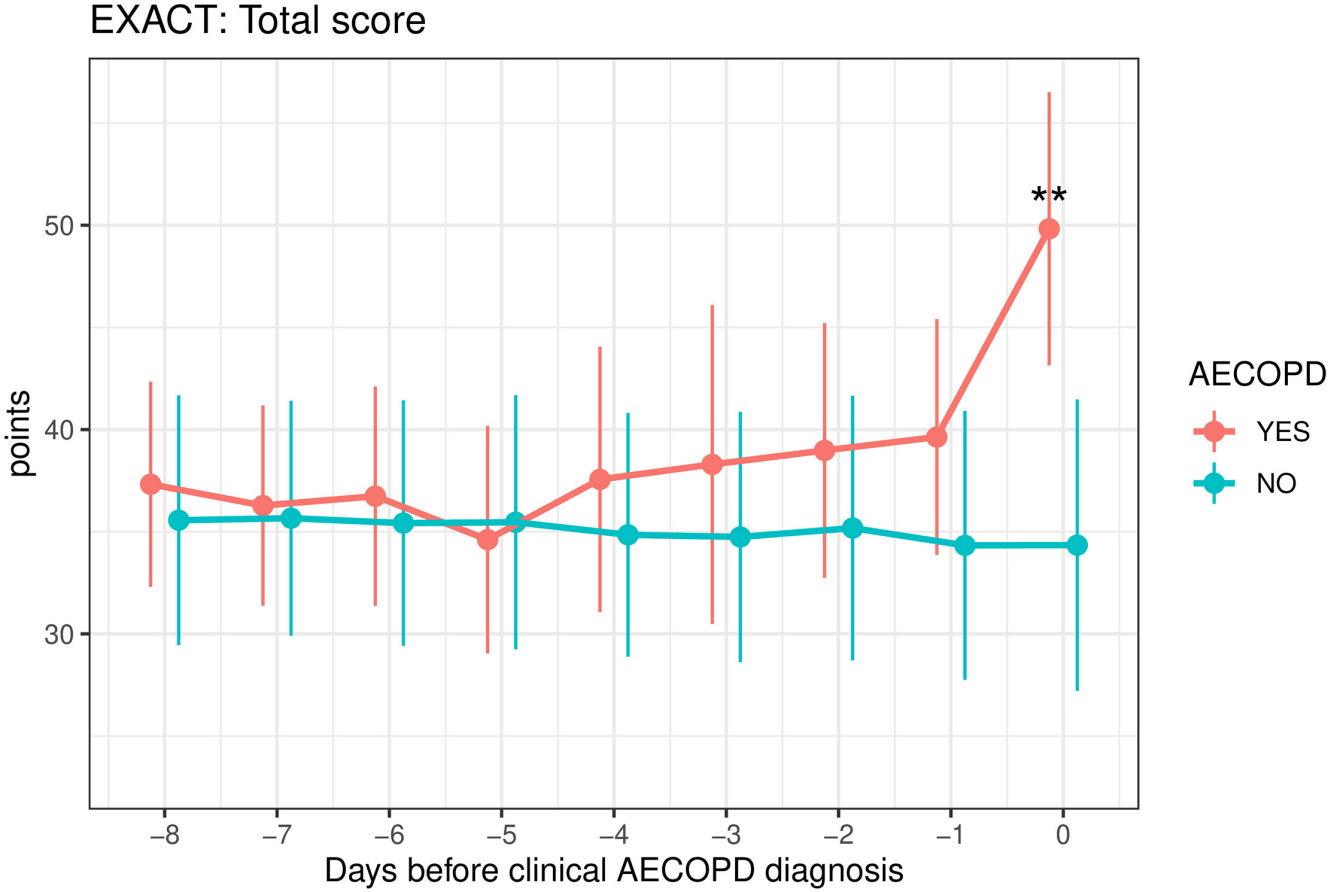
Prognostic value of the EXAcerbations of Chronic Obstructive Pulmonary Disease Tool (EXACT) total score in recognising acute COPD exacerbations. Day 0 on the *x*‐axis represents the day of the clinical AECOPD diagnosis. Values are presented as bootstrap mean ± bootstrap 95% CI. **p < 0.01.

**FIGURE 3:**
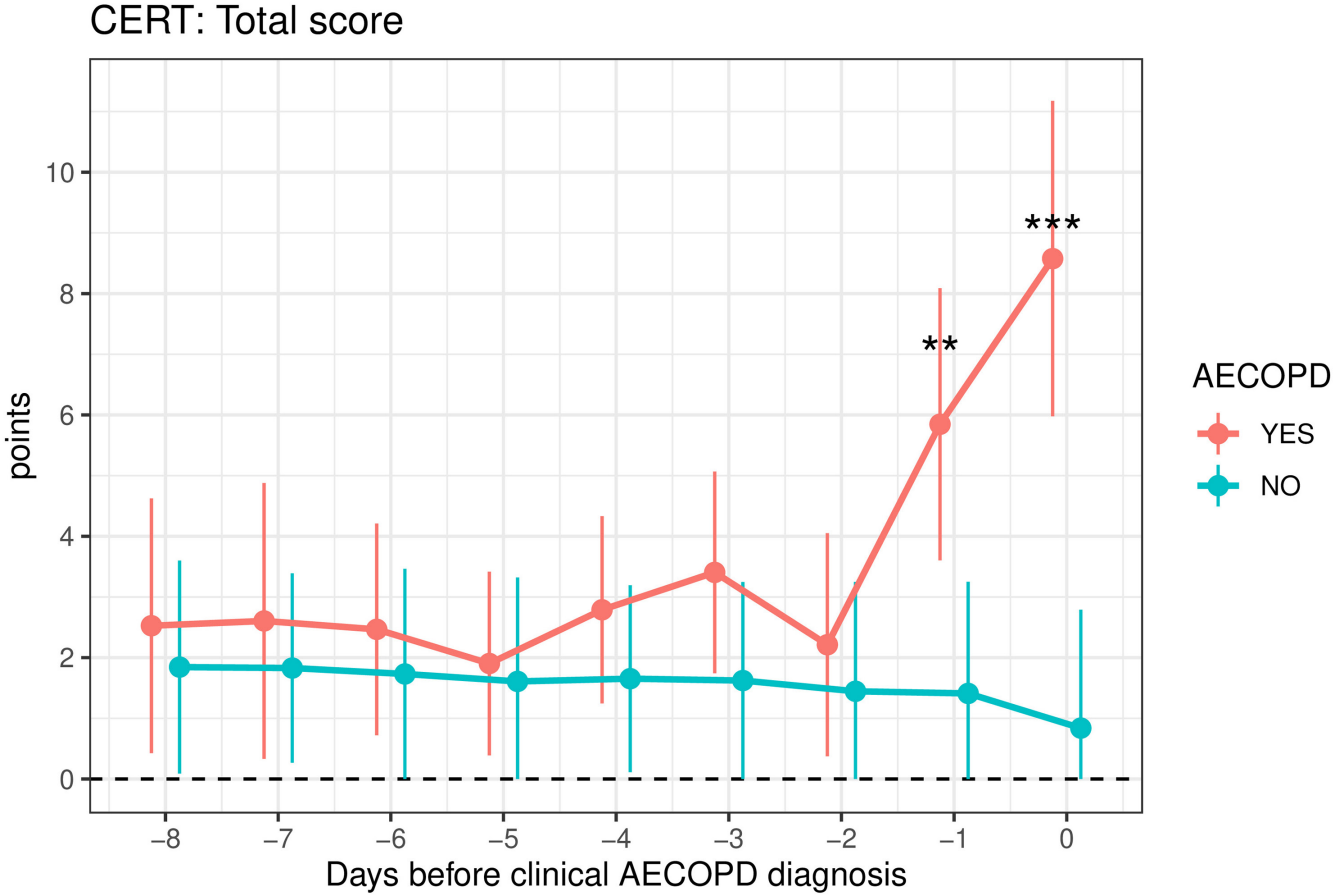
Prognostic value of the COPD Exacerbation Recognition Tool (CERT) total score in recognising acute COPD exacerbations. Day 0 on the *x*‐axis represents the day of the clinical AECOPD diagnosis. Values are presented as bootstrap mean ± bootstrap 95% CI. **p < 0.01, ***p < 0.001.

**FIGURE 4:**
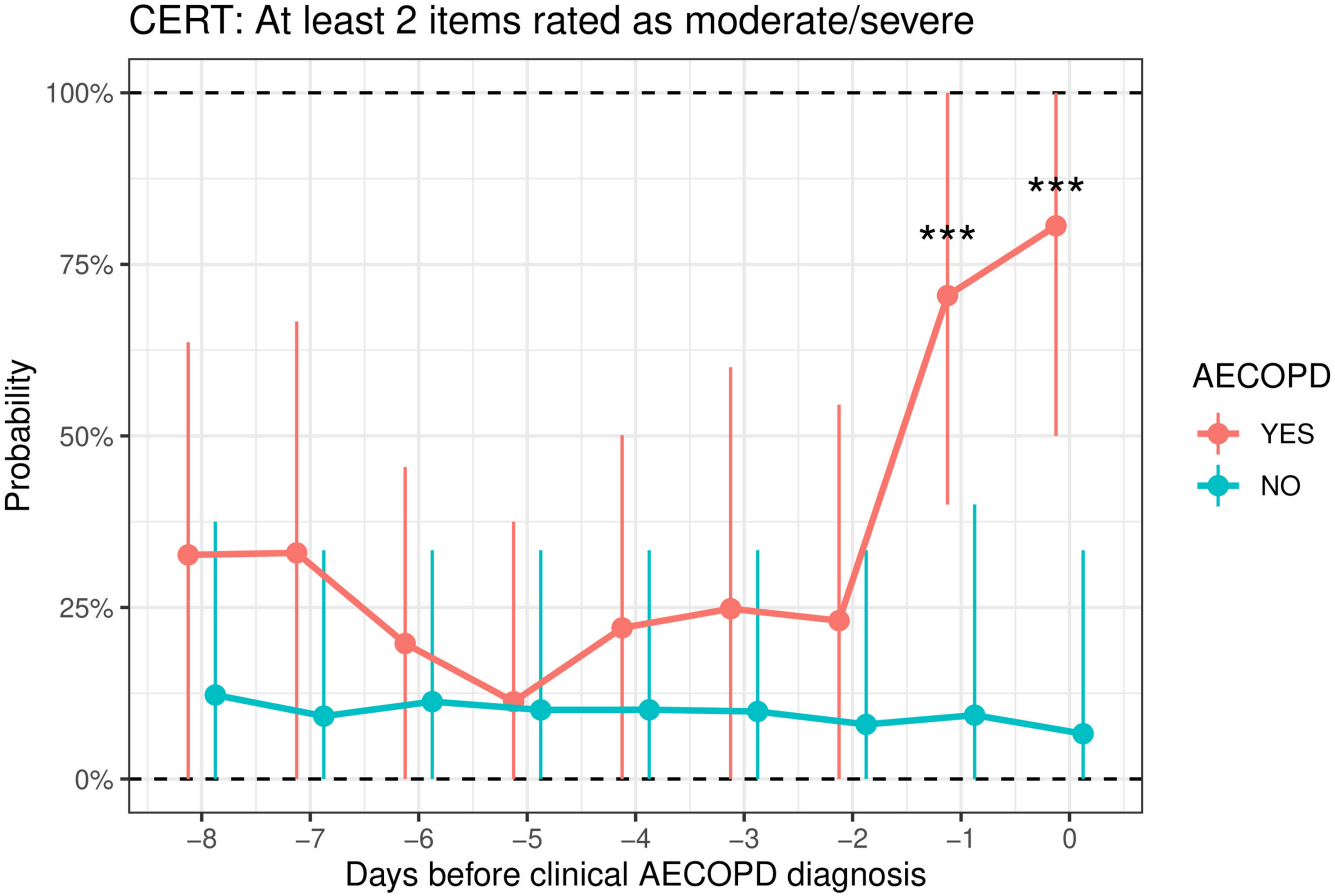
Prognostic value of the COPD Exacerbation Recognition Tool (CERT) to recognise an acute COPD exacerbation when at least 2 CERT items were rated as moderate or severe. Day 0 on the *x*‐axis represents the day of the clinical AECOPD diagnosis. Values are presented as bootstrap mean ± bootstrap 95% CI. ***p < 0.001.

On the day of AECOPD diagnosis, both EXACT and CERT scores were markedly higher in patients with a confirmed AECOPD than in those without (Table S1; Figures 2, 3, 4). EXACT total scores averaged 49.8 versus 34.3 points (*p* = 0.002) while CERT total scores averaged 8.6 versus 0.8 (*p* < 0.001). Moreover, 80.6% of patients with an AECOPD met the CERT ‘≥ 2 items rated as moderate/severe’ criterion compared to 6.6% of patients without an exacerbation (*p* = 0.009).

In ROC analyses, all tools demonstrated excellent discrimination (Table S1). EXACT achieved an AUC of 0.90, and CERT performed nearly as well with an AUC of 0.88 for the total score and 0.87 for the ‘≥ 2 items’ rule. At optimal thresholds, an EXACT cutoff of 44.9 points yielded 87% sensitivity, 93% specificity, 94% PPV, and 89% NPV; a CERT cutoff of 4.5 points provided 81% sensitivity, 95% specificity, 96% PPV, and 77% NPV. Detailed predictive values for all cut‐offs are available in Table S2.

### 4.2 Prediction of AECOPD One Day *Before* Clinical Diagnosis

One day before clinical AECOPD confirmation, CERT scores were significantly higher in patients who subsequently experienced an AECOPD than in those who did not (mean CERT total 5.9 vs. 1.4; *p* = 0.006), and 70.4% of future exacerbators met the CERT ‘≥ 2 items’ criterion versus 9.3% of non‐exacerbators (*p* = 0.009). EXACT scores trended higher in future exacerbators (39.6 vs. 34.3) but did not reach statistical significance (*p* = 0.186).

**FIGURE 5:**
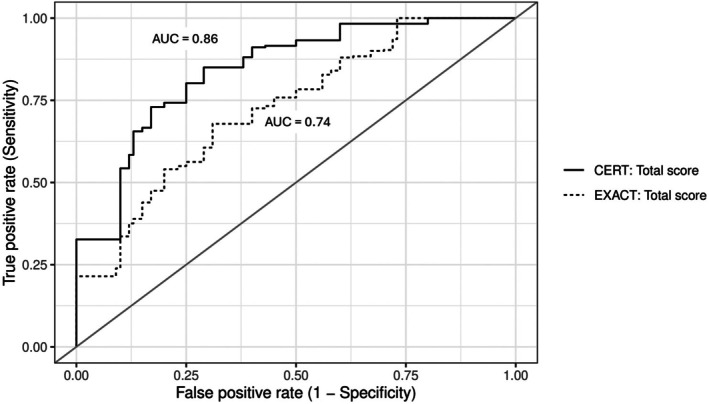
Receiver operating characteristic (ROC) curves showing the predictive performance of the Chronic Obstructive Pulmonary Disease Exacerbation Recognition Tool (CERT) and the Exacerbations of Chronic Pulmonary Disease Tool (EXACT) one day before the clinical diagnosis of an acute COPD exacerbation. The curves represent the averaged results from 1000 bootstrap iterations per tool. AUC = area under the curve.

In ROC analyses, CERT total score outperformed EXACT (AUC 0.86 vs. 0.74), (Figure 5) and the CERT ‘≥ 2 items’ rule also showed strong discrimination (AUC 0.81). At their optimal cut‐offs (Table S2), EXACT (37.9 points) achieved 80% sensitivity and 75% specificity, whereas CERT total score (2.75 points) delivered both higher sensitivity (83%) and specificity (86%). The CERT ‘≥ 2 items’ criterion had a higher specificity at 91%, with a modest trade‐off in sensitivity (70%). Detailed predictive values (PPV/NPV) for all thresholds are provided in Table S2.

## 5 Discussion

This comparative analysis of EXACT and CERT for detecting and predicting AECOPD revealed several key findings. First, on the day of clinical diagnosis, both tools demonstrated a similar strong discriminative ability between exacerbators and non‐exacerbators (AUC between 0.87 and 0.90). However, the most striking result was that CERT detected a significant worsening of symptoms one day before the clinical AECOPD diagnosis, whereas EXACT did not. This finding is clinically significant, as early recognition and prompt treatment of AECOPDs can improve outcomes [3].

Jones et al. [12] originally developed CERT as a concise, patient‐centered checklist targeting five core symptoms associated with AECOPD, derived from patient interviews and expert consensus. It was designed as an educational tool to help patients recognise symptomatic deterioration and is typically used only when worsening is perceived. In a sensitivity analysis during CERT's development, they used data from 150 stable outpatients and 48 patients with known exacerbations, demonstrating 91.8% sensitivity and 100% specificity for identifying an AECOPD on the day of diagnosis [12]. However, that study was retrospective, using data that had been used in CERT's development whereas our study had a prospective design.

To allow for a direct comparison with the EXACT diary, the CERT was applied daily, which deviated from its intended episodic use. Administering CERT in a daily diary format may influence how patients anchor their responses, leading them to focus on the current day, develop response habituation through repetition, and lose opportunities for clinician clarification. Nevertheless, CERT's strong performance highlights its potential to support the early recognition of AECOPD. CERT detected deterioration one day before diagnosis, suggesting that the guidance about when to seek medical assessment is sufficient to capture early symptom onset. We therefore recommend maintaining CERT's original episodic use and instructions in clinical practice, while ensuring that patients are aware they should complete the CERT when they perceive a worsening of their symptoms.

Whereas the CERT's symptom‐focused design, prompts patients to notice sudden changes, such as increased sputum or breathlessness, the EXACT approach is impact‐based (e.g., ‘Did your cough keep you from sleeping?’), which may lag behind rapid symptom shifts. Also, practical usability considerations further distinguish CERT and EXACT. The complexity of EXACT and its 14‐item daily diary format (completion time around 5 min) limit its feasibility for widespread adoption outside a research setting [17]. Furthermore, it is also copyrighted and requires licensing for any usage. In contrast, the CERT is freely available, uses plain language (available in > 40 languages) with pictograms, and is easy and quick to administer (< 1 min). This may support higher compliance.

The CERT was not originally designed to be scored. It simply established a binary criterion: ‘If at least two items are rated as moderate or severe, seek medical attention’. For this study, the severity levels associated with the symptoms were scored on a 0–5 scale, providing a total range of 0–15. Both methods showed strong discrimination in our cohort (day of clinical AECOPD diagnosis: AUC 0.87–0.88; one day prior: AUC 0.81–0.86). A total score of ≥ 3 points yielded 83% sensitivity and 86% specificity for predicting AECOPD one day before diagnosis, while the binary approach had 70% sensitivity and 91% specificity. CERT's concise format and straightforward thresholds make it well‐suited for patient self‐monitoring, with the binary method being especially user‐friendly (because no calculations are required), whereas the total score may be more applicable for research settings. This comparison of total versus binary scoring approaches was conducted within the context of this validation study that used CERT as a daily diary to test the responsiveness of its items to early symptom changes. The study design does not reflect CERT's original purpose as an episodic tool intended for use when patients perceive symptom worsening. Consequently, the sensitivity and specificity values reported here describe the tool's performance under daily diary conditions and do not establish recommendations for clinical scoring thresholds or everyday application. Further work is needed to determine optimal scoring and timing strategies under routine, episodic use.

The clinical implications of our findings are multifold. Integrating CERT into patient education curricula could empower patients to recognise the onset of symptoms and seek timely medical advice. This could potentially reduce treatment delays, as delays of more than 24 h have been associated with a twofold increase in the odds of hospitalisation and worse outcomes [18]. Our findings suggest that CERT's educational checklist may further bridge the gap in patients' perceptions of AECOPD symptoms, which are very heterogeneous [9]. Jones et al. highlight key barriers to exacerbation reporting, including low symptom awareness and attitudes toward seeking care [19]. By giving patients clear, actionable symptom descriptors, CERT may potentially improve patient empowerment and subsequently AECOPD reporting.

For future applications, CERT could also be implemented as an electronic version with automated alerts on telemedicine platforms or in digital disease management programs. CERT entries that meet predefined thresholds could prompt notifications to patients and/or care teams, enabling them to intervene proactively. This approach aligns with the evidence that supports the effectiveness of telemonitoring in improving AECOPD reporting and reducing the worsening of symptoms over time [20, 21].

A major strength of this study is its inpatient rehabilitation setting, which enables prompt clinical assessment and early AECOPD diagnosis through daily supervision by pulmonologists who were blinded to EXACT/CERT ratings. Unlike outpatient studies where EXACT often serves as a surrogate due to delayed medical contact settings [17], our setting ensured timely, physician‐confirmed diagnoses of AECOPD, reducing diagnostic uncertainty and strengthening the reference standard for AECOPD detection. To further enhance diagnostic rigour, these clinical assessments were complemented by comprehensive objective measures including spirometry, blood gas analyses, inflammatory markers (e.g., CRP) performed according to the PACE trial protocol [13]. This multiparametric approach ensures a robust, validated gold standard AECOPD diagnosis beyond symptom‐based evaluations alone.

Despite these strengths, several limitations warrant consideration. First, the small number of AECOPD events (*n* = 12) in a single‐center setting limits the study's generalizability. Although bootstrapping and matched control analyses mitigate sample size constraints, they cannot fully replace larger prospective validation. Second, patient understanding is crucial: eight participants were excluded from CERT analyses due to consistently high scores, which suggest misinterpretation. Patients seemed to rate their current symptom burden similarly to that in the COPD Assessment Test (CAT) [22], rather than deterioration in symptoms. This highlights the need for thorough instruction and potential periodic retraining. However, overall inaccuracy was similar between EXACT and CERT, making systematic bias between the tools driven by differing patient characteristics unlikely. Lastly, this study used the CERT instruction to indicate the extent of the current symptom deterioration ‘in the last two days’ instead of ‘compared to your usual state’ from the original validation study. However, the original validation study found no difference in responses when patients compared their symptoms to either their baseline or their current state during an exacerbation [12]. Therefore, the original wording of ‘compared to your usual state’ could remain.

Future studies should include multicenter trials to validate CERT's predictive thresholds across diverse patient populations and settings, including outpatient and home‐based groups. Comparative studies evaluating hybrid strategies, such as combining CERT's early warning signals with objective physiological metrics (e.g., respiratory rate, SpO_2_, and wearable sensors), could enhance specificity and reduce false positives. Integrating CERT into existing electronic health records and digital platforms, would streamline data flow and automate risk stratification [23]. Additionally, qualitative research to refine item wording and ensure cultural adaptability will support global dissemination. For example, the Japanese CERT version (CERT‐J) includes noting sputum colour changes [24].

In conclusion, CERT demonstrates robust early predictive validity for AECOPD one day prior to clinical diagnosis, outperforming EXACT during this critical window. Its concise, patient‐friendly format lends itself to routine monitoring and telemedicine integration. Future multicenter studies and digital implementations are essential to confirm CERT's broader utility and optimise COPD management.

## Author Contributions

**Rainer Gloeckl:** conceptualization, writing – original draft, data curation, writing – review and editing. **Paul W. Jones:** conceptualization, writing – review and editing, supervision. **Daniela Kroll:** conceptualization, data curation, writing – review and editing. **Inga Jarosch:** conceptualization, data curation, writing – review and editing. **Tessa Schneeberger:** conceptualization, data curation, writing – review and editing. **Jing Claussen:** conceptualization, writing – review and editing. **Paul Schmidt:** formal analysis, conceptualization, writing – review and editing, visualization. **Claus F. Vogelmeier:** conceptualization, writing – review and editing. **Klaus Kenn:** conceptualization, writing – review and editing. **Andreas Rembert Koczulla:** conceptualization, writing – review and editing, supervision.

## Funding

The PACE study (ID 212667) is a supported, collaborative study funded by GSK. However, GSK did not provide additional funding for the amendment concerning the inclusion of the CERT.

## Ethics Statement

This study was performed in accordance with the Declaration of Helsinki. This human study was approved by the Ethics Committee of the Philipps University Marburg, Germany—approval: 61/19 Amendment #2. The study's clinical trial registration number is NCT04140097 registered with www.clinicaltrials.gov. Participant registration took place in Jul–2023. All adult participants provided written informed consent to participate in this study.

## Conflicts of Interest

R.G. and P.J. are members of the international CERT Governance Board. J.C. is an employee of GlaxoSmithKline. P.J. reports being a part‐time contractor with GlaxoSmithKline, and owning GlaxoSmithKline stocks and shares outside the submitted work. All other authors did not report any conflicts of interest.

## Supporting information


**Table S1:** Values for the total score of the Exacerbations of Chronic Pulmonary Disease Tool (EXACT), and the COPD Exacerbation Recognition Tool (CERT), as well as the rating when at least two items were scored as moderate or severe several days before and on the day of the clinical diagnosis of an acute COPD exacerbation (AECOPD), in *n* = 12 with AECOPD and *n* = 12 matched COPD patients without AECOPD.
**Table S2:** Prognostic quality of the total scores of the Exacerbations of Chronic Pulmonary Disease Tool (EXACT), and the COPD Exacerbation Recognition Tool (CERT), as well as the rating when at least two items were scored as moderate or severe several days before and on the day of the clinical diagnosis of an acute COPD exacerbation (AECOPD), in *n* = 12 with AECOPD and *n* = 12 matched COPD patients without AECOPD. Data are presented as value and 95% CI.

## Data Availability

De‐identified individual participant data and a data dictionary defining each field in the set can be made available to others on approval of a written request to the corresponding author. The request will be evaluated by a committee formed by a subset of co‐authors to determine the research value. A data‐sharing agreement will be needed.
