# Supplementary material for: Can Patient Education Lead the Way? Head‐To‐Head Comparison of EXACT and CERT for Early Recognition of Acute COPD Exacerbations
Source: Respirology. 2025 Nov 30;31(3):256–64. doi: 10.1002/resp.70170 (PMC12963703; doi:10.1002/resp.70170)
Supplement: Supplementary file 1 — Table S1: Values for the total score of the Exacerbations of Chronic Pulmonary Disease Tool (EXACT), and the COPD Exacerbation Recognition Tool (CERT), as well as the rating when at least two items were scored as moderate or severe several days before and on the day of the clinical diagnosis of an acute COPD exacerbation (AECOPD), in n = 12 with AECOPD and n = 12 matched COPD patients without AECOPD. Table S2: Prognostic quality of the total scores of the Exacerbations of Chronic Pulmonary Disease Tool (EXACT), and the COPD Exacerbation Recognition Tool (CERT), as well as the rating when at least two items were scored as moderate or severe several days before and on the day of the clinical diagnosis of an acute COPD exacerbation (AECOPD), in n = 12 with AECOPD and n = 12 matched COPD patients without AECOPD. Data are presented as value and 95% CI. [file RESP-31-256-s001.docx]

**- - -Online Supplement - - -**

**Title: Can patient education lead the way? Head-to-head comparison of EXACT and CERT for early recognition of acute COPD exacerbations**

**Authors:** Rainer Gloeckl, Paul W. Jones, Daniela Kroll, Inga Jarosch, Tessa Schneeberger,

Jing Claussen, Paul Schmidt, Claus F. Vogelmeier, Klaus Kenn, Rembert Koczulla

**Table S1. Values for the total score of the Exacerbations of Chronic Pulmonary Disease Tool (EXACT), and the COPD Exacerbation Recognition Tool (CERT), as well as the rating when at least two items were scored as moderate or severe several days before and on the day of the clinical diagnosis of an acute COPD exacerbation (AECOPD), in n=12 with AECOPD and n=12 matched COPD patients without AECOPD.**

|  | **EXACT**  **total score** | | | **CERT**  **total score** | | | **CERT**  **at least 2 items moderate/severe** | | |  |
| --- | --- | --- | --- | --- | --- | --- | --- | --- | --- | --- |
|  | **AECOPD**  **NO**  *EMM (SE) 95%CI* | **AECOPD**  **YES**  *EMM (SE) 95%CI* | **Difference**  **YES – NO** *(SE) 95%CI*  *p-value* | **AECOPD NO**  *EMM (SE) 95%CI* | **AECOPD YES**  *EMM (SE) 95%CI* | **Difference**  **YES – NO** *(SE) 95%CI*  *p-value* | **AECOPD NO**  *Prob (%) 95%CI* | **AECOPD YES**  *Prob (%) 95%CI* | **Odds Ratio**  *95%CI*  *p-value* | |
| Day of clinical AECOPD diagnosis | 34.35 (3.64) 27.22; 41.47 | 49.83 (3.41) 43.14; 56.52 | 15.48 (4.75) 6.16; 24.8 **p = 0.002** | 0.84 (1) -1.11; 2.79 | 8.58 (1.33) 5.98; 11.18 | 7.74 (1.78) 4.24; 11.23 **p < 0.001** | 6.6 0; 33.3 | 80.6 50; 100 | 19.13 2.01; 62.7 **p = 0.009** | |
| 1 day before AECOPD diagnosis | 34.33 (3.36) 27.75; 40.91 | 39.64 (2.94) 33.87; 45.41 | 5.3 (4.13) -2.8; 13.4 p = 0.186 | 1.41 (0.94) -0.43; 3.25 | 5.85 (1.14) 3.6; 8.09 | 4.44 (1.58) 1.35; 7.52 **p = 0.006** | 9.3 0; 40 | 70.4 40; 100 | 14.58 1.67; 50 **p = 0.009** | |
| 2 days before AECOPD diagnosis | 35.18 (3.3) 28.71; 41.65 | 38.98 (3.18) 32.75; 45.21 | 3.8 (4.06) -4.15; 11.75 p = 0.342 | 1.45 (0.92) -0.36; 3.25 | 2.21 (0.94) 0.38; 4.05 | 0.77 (1.28) -1.74; 3.28 p = 0.536 | 8 0; 33.3 | 23.1 0; 54.5 | 1.91 < 0.001; 6.4 p = 0.786 | |
| 3 days before AECOPD diagnosis | 34.75 (3.13) 28.62; 40.87 | 38.3 (3.98) 30.5; 46.09 | 3.55 (4.86) -5.98; 13.08 p = 0.462 | 1.62 (0.83) 0; 3.25 | 3.4 (0.85) 1.74; 5.07 | 1.78 (1.22) -0.61; 4.17 p = 0.138 | 9.8 0; 33.3 | 24.8 0; 60 | 1.97 < 0.001; 7.19 p = 0.738 | |
| 4 days before AECOPD diagnosis | 34.85 (3.04) 28.89; 40.81 | 37.56 (3.31) 31.07; 44.05 | 2.71 (4.18) -5.48; 10.9 p = 0.516 | 1.65 (0.79) 0.11; 3.19 | 2.79 (0.79) 1.25; 4.33 | 1.14 (1.19) -1.2; 3.47 p = 0.318 | 10.1 0; 33.3 | 22 0; 50.1 | 1.85 < 0.001; 7.36 p = 0.803 | |
| 5 days before AECOPD diagnosis | 35.46 (3.17) 29.25; 41.68 | 34.61 (2.84) 29.04; 40.18 | -0.85 (3.77) -8.24; 6.54 p = 0.838 | 1.61 (0.87) -0.11; 3.32 | 1.9 (0.77) 0.39; 3.42 | 0.29 (1.18) -2.02; 2.61 p = 0.764 | 10.1 0; 33.3 | 11.3 0; 37.5 | 0.92 < 0.001; 3.6 p = 0.707 | |
| 6 days before AECOPD diagnosis | 35.42 (3.07) 29.41; 41.43 | 36.74 (2.74) 31.37; 42.11 | 1.32 (3.64) -5.82; 8.45 p = 0.732 | 1.73 (0.88) 0; 3.47 | 2.47 (0.89) 0.72; 4.21 | 0.74 (1.31) -1.83; 3.3 p = 0.558 | 11.3 0; 33.3 | 19.7 0; 45.5 | 1.75 < 0.001; 6.45 p = 0.88 | |
| 7 days before AECOPD diagnosis | 35.66 (2.93) 29.91; 41.42 | 36.28 (2.5) 31.39; 41.18 | 0.62 (3.4) -6.05; 7.29 p = 0.84 | 1.83 (0.8) 0.27; 3.39 | 2.6 (1.16) 0.33; 4.88 | 0.78 (1.48) -2.12; 3.67 p = 0.588 | 9.2 0; 33.3 | 33 0; 66.7 | 4.03 < 0.001; 14 p = 0.386 | |
| 8 days before AECOPD diagnosis | 35.56 (3.12) 29.45; 41.68 | 37.32 (2.56) 32.31; 42.34 | 1.76 (3.53) -5.16; 8.68 p = 0.6 | 1.84 (0.9) 0.09; 3.6 | 2.53 (1.07) 0.43; 4.62 | 0.68 (1.45) -2.16; 3.52 p = 0.632 | 12.2 0; 37.5 | 32.7 0; 63.6 | 3.5 < 0.001; 12.19 p = 0.371 | |

**Table S2. Prognostic quality of the total scores of the Exacerbations of Chronic Pulmonary Disease Tool (EXACT), and the COPD Exacerbation Recognition Tool (CERT), as well as the rating when at least two items were scored as moderate or severe several days before and on the day of the clinical diagnosis of an acute COPD exacerbation (AECOPD),** **in n=12 with AECOPD and n=12 matched COPD patients without AECOPD. Data are presented as value and 95%CI.**

|  | **Score** | **AUC** | **Threshold** | **Accuracy** | **Sensitivity** | **Specificity** | **Pos Pred Value** | **Neg Pred Value** |
| --- | --- | --- | --- | --- | --- | --- | --- | --- |
| day of clinical  AECOPD diagnosis | EXACT  total score | 0.9 0.7; 1 | 44.93 33.94; 56.00 | 0.9 0.74; 1 | 0.87 0.59; 1 | 0.93 0.68; 1 | 0.94 0.72; 1 | 0.89 0.66; 1 |
|  | CERT  total score | 0.88 0.72; 1.0 | 4.51 0.27; 8.8 | 0.86 0.7; 1.0 | 0.81 0.54; 1.0 | 0.95 0.76; 1.0 | 0.96 0.83; 1.0 | 0.77 0.51; 1.0 |
|  | CERT  „2 item approach“ | 0.87 0.71; 1 | **-** | 0.86 0.69; 1 | 0.81 0.56; 1 | 0.93 0.73; 1 | 0.95 0.82; 1 | 0.76 0.49; 1 |
| 1 day  before  AECOPD diagnosis | EXACT  total score | 0.74 0.47; 1.00 | 37.92 24.89; 50.94 | 0.77 0.58; 0.96 | 0.8 0.35; 1.00 | 0.75 0.28; 1.00 | 0.84 0.56; 1.00 | 0.82 0.45; 1.00 |
|  | CERT  total score | 0.86 0.67; 1.0 | 2.75 0; 5.9 | 0.84 0.68; 1.0 | 0.83 0.55; 1.0 | 0.86 0.59; 1.0 | 0.91 0.72; 1.0 | 0.8 0.54; 1.0 |
|  | CERT  „2 item approach“ | 0.81 0.61; 1.00 | - | 0.79 0.59; 0.98 | 0.7 0.41; 1.00 | 0.91 0.67; 1.00 | 0.92 0.73; 1.00 | 0.68 0.43; 0.93 |
| 2 days  before  AECOPD diagnosis | EXACT  total score | 0.65 0.37; 0.92 | 40.13 25.11; 55.15 | 0.7 0.5; 0.90 | 0.62 0.14; 1.00 | 0.81 0.36; 1.00 | 0.88 0.62; 1.00 | 0.65 0.31; 0.98 |
|  | CERT  total score | 0.67 0.43; 0.92 | 1.1 0; 3.72 | 0.71 0.51; 0.91 | 0.72 0.32; 1.00 | 0.7 0.33; 1.00 | 0.77 0.52; 1.00 | 0.7 0.39; 1.00 |
|  | CERT  „2 item approach“ | 0.58 0.4; 0.75 | - | 0.53 0.34; 0.73 | 0.23 0; 0.51 | 0.92 0.71; 1.00 | 0.79 0.26; 1.00 | 0.49 0.31; 0.66 |
| 3 days  before  AECOPD diagnosis | EXACT  total score | 0.71 0.41; 1.00 | 40.41 26.88; 53.93 | 0.77 0.57; 0.97 | 0.75 0.31; 1.00 | 0.78 0.37; 1.00 | 0.82 0.49; 1.00 | 0.81 0.52; 1.00 |
|  | CERT  total score | 0.73 0.49; 0.98 | 2.33 0.61; 4.06 | 0.78 0.58; 0.97 | 0.74 0.41; 1.00 | 0.81 0.54; 1.00 | 0.81 0.55; 1.00 | 0.77 0.54; 1.00 |
|  | CERT  „2 item approach“ | 0.57 0.38; 0.77 | - | 0.57 0.37; 0.78 | 0.25 0; 0.56 | 0.9 0.69; 1.00 | 0.71 0.12; 1.00 | 0.55 0.37; 0.72 |
| 4 days  before  AECOPD diagnosis | EXACT  total score | 0.66 0.38; 0.93 | 38.35 21.07; 55.64 | 0.71 0.54; 0.89 | 0.71 0.19; 1.00 | 0.72 0.21; 1.00 | 0.8 0.49; 1.00 | 0.76 0.41; 1.00 |
|  | CERT  total score | 0.71 0.47; 0.95 | 1.59 0; 3.81 | 0.73 0.54; 0.91 | 0.71 0.34; 1.00 | 0.76 0.43; 1.00 | 0.78 0.52; 1.00 | 0.72 0.44; 0.99 |
|  | CERT  „2 item approach“ | 0.56 0.39; 0.73 | - | 0.54 0.35; 0.74 | 0.22 0; 0.50 | 0.9 0.69; 1.00 | 0.71 0.12; 1.00 | 0.51 0.35; 0.68 |
| 5 days  before  AECOPD diagnosis | EXACT  total score | 0.58 0.32; 0.84 | 35.56 24.66; 46.47 | 0.68 0.5; 0.86 | 0.73 0.27; 1.00 | 0.63 0.19; 1.00 | 0.73 0.47; 0.98 | 0.71 0.39; 1.00 |
|  | CERT  total score | 0.66 0.42; 0.90 | 0.85 0; 2.81 | 0.7 0.52; 0.89 | 0.75 0.37; 1.00 | 0.66 0.33; 1.00 | 0.71 0.47; 0.95 | 0.75 0.46; 1.00 |
|  | CERT  „2 item approach“ | 0.51 0.36; 0.65 | - | 0.5 0.33; 0.67 | 0.11 0; 0.32 | 0.9 0.7; 1.00 | 0.53 0; 1.00 | 0.5 0.35; 0.65 |
| 6 days  before  AECOPD diagnosis | EXACT  total score | 0.63 0.37; 0.89 | 35.72 25.78; 46.65 | 0.71 0.53; 0.88 | 0.77 0.35; 1.00 | 0.63 0.2; 1.00 | 0.74 0.51; 0.98 | 0.75 0.43; 1.00 |
|  | CERT  total score | 0.69 0.46; 0.93 | 1.35 0; 4.42 | 0.72 0.53; 0.90 | 0.73 0.33; 1.00 | 0.71 0.37; 1.00 | 0.75 0.51; 0.99 | 0.74 0.48; 1.00 |
|  | CERT  „2 item approach“ | 0.54 0.38; 0.70 | - | 0.53 0.36; 0.70 | 0.2 0; 0.45 | 0.89 0.69; 1.00 | 0.66 0.07; 1.00 | 0.5 0.37; 0.64 |
| 7 days  before  AECOPD diagnosis | EXACT  total score | 0.61 0.34; 0.89 | 38.42 23.13; 53.70 | 0.7 0.52; 0.88 | 0.65 0.2; 1.00 | 0.75 0.29; 1.00 | 0.76 0.45; 1.00 | 0.73 0.46; 1.00 |
|  | CERT  total score | 0.75 0.53; 0.98 | 2.58 0; 5.84 | 0.76 0.59; 0.94 | 0.71 0.35; 1.00 | 0.83 0.53; 1.00 | 0.83 0.58; 1.00 | 0.75 0.52; 0.99 |
|  | CERT  „2 item approach“ | 0.62 0.44; 0.80 | - | 0.61 0.42; 0.80 | 0.33 0.01; 0.65 | 0.91 0.71; 1.00 | 0.8 0.37; 1.00 | 0.56 0.41; 0.72 |
| 8 days  before  AECOPD diagnosis | EXACT  total score | 0.63 0.36; 0.89 | 36.38 22.45; 50.32 | 0.7 0.52; 0.88 | 0.76 0.34; 1.00 | 0.65 0.23; 1.00 | 0.72 0.47; 0.98 | 0.76 0.47; 1.00 |
|  | CERT  total score | 0.69 0.45; 0.92 | 2.65 0.46; 4.85 | 0.73 0.55; 0.91 | 0.64 0.31; 0.98 | 0.81 0.55; 1.00 | 0.78 0.53; 1.00 | 0.71 0.5; 0.92 |
|  | CERT  „2 item approach“ | 0.6 0.42; 0.79 | - | 0.6 0.41; 0.79 | 0.33 0.01; 0.64 | 0.88 0.66; 1.00 | 0.74 0.29; 1.00 | 0.57 0.41; 0.73 |
